# A qualitative study of old patients’ experiences of the quality of the health services in hospital and 30 days after hospitalization

**DOI:** 10.1186/s12913-020-05303-5

**Published:** 2020-05-20

**Authors:** Ingvild Lilleheie, Jonas Debesay, Asta Bye, Astrid Bergland

**Affiliations:** 1 Department of Physiotherapy, Faculty of Health Sciences, Oslo Metropolitan University, Oslo, Norway; 2 Department of Nursing and Health Promotion, Faculty of Health Sciences, Oslo Metropolitan University, Oslo, Norway; 3 European Palliative Care Research Centre (PRC), Department of Oncology, Oslo University Hospital and Institute of Clinical Medicine, University of Oslo, Oslo, Norway; 4 Department of Physiotherapy, Faculty of Health Sciences, Oslo Metropolitan University, Oslo, Norway

**Keywords:** Healthcare, Elderly, Quality of care, Patient-centered

## Abstract

**Background:**

The number of people aged 80 years and above is projected to triple over the next 30 years. People in this age group normally have at least two chronic conditions. The impact of multimorbidity is often significantly greater than expected from the sum of the effects of each condition. The World Health Organization has indicated that healthcare systems must prepare for a change in the focus of clinical care for older people. The World Health Organization (WHO) defines healthcare quality as care that is effective, efficient, integrated, patient centered, equitable and safe. The degree to which healthcare quality can be defined as acceptable is determined by services’ ability to meet the needs of users and adapt to patients’ expectations and perceptions.

**Method:**

We took a phenomenological perspective to explore older patients’ subjective experiences and conducted semistructured individual interviews. Eighteen patients (aged from 82 to 100 years) were interviewed twice after discharge from hospital. The interview transcriptions were analyzed thematically.

**Results:**

The patients found their meetings with the health service to be complex and demanding. They reported attempting to restore a sense of security and meaning in everyday life, balancing their own needs against external requirements. Five overarching themes emerged from the interviews: hospital stay and the person behind the diagnosis, poor communication and coordination, life after discharge, relationship with their next of kin, and organizational and systemic determinants.

**Conclusion:**

According to the WHO, to deliver quality healthcare, services must include all six of the dimensions listed above. Our findings show that they do not. Healthcare focused on measurable values and biomedical inquiries. Few opportunities for participation, scant information and suboptimal care coordination left the patients with a feeling of being in limbo, where they struggled to find balance in their everyday life. Further work must be done to ensure that integrated services are provided without a financial burden, centered on the needs and rights of older people.

## Background

According to the United Nations (UN), the number of people aged 80 and above is projected to triple over the next 30 years [1]. The norm in this age group is that people have at least two chronic conditions (multimorbidity) [2]. The impact of multimorbidity on an older person’s capacity, healthcare utilization and their costs of care is often significantly greater than might be expected from the sum of the effects of each condition. Therefore, the World Health Organization’s (WHO) *World Report on Aging and Health* [3] urges healthcare systems to prepare for a fundamental change in the focus of clinical care for older people. In recent years, most Western countries have transferred responsibility for tasks previously carried out in hospitals to municipalities. The result of this policy is decreased hospitalization time in all OECD (Organisation for Economic Co-operation and Development) countries [4]. As the average length of stay declines, two consequences have appeared: elderly people are discharged from the hospital “quicker and sicker” than previously [5, 6], and the healthcare provider has less time to coordinate services across settings and to prepare the patients for the home situation [7]. The WHO defines healthcare quality as care that is effective, efficient, integrated, patient centered, equitable and safe [8]. The degree to which healthcare quality can be defined as acceptable is strongly dependent on service providers’ ability to meet the needs of their users [9] and adapt to patients’ expectations and perceptions [10].

The concept of patient centeredness and patient participation is defined as “a patient’s rights and opportunities to influence and engage in the decision making about his care through a dialogue attuned to his preferences, potential and a combination of his experiential and the professional’s expert knowledge” [11]. Successful patient participation is associated with satisfaction with healthcare services [12], better treatment outcomes [13], shorter institutional stays [14] and fewer readmissions [15]. The transition between levels of the healthcare system and the period subsequent to hospital discharge is critical for elderly patients [16, 17]. Therefore, readmissions within 30 days after hospital discharge, referred to as “30-day rehospitalizations,” are frequent adverse outcomes for this population [18, 19] with deleterious consequences, including loss of autonomy, increased mortality [20, 21] and high socioeconomic costs [22, 23]. All factors related to health quality based on the WHO definition.

The key challenge in achieving good quality healthcare for older patients is to adapt it to the needs of older people—as perceived by themselves. The patients’ perspective on their care journey between levels of the healthcare system may provide valuable information to guide quality improvement in health services [24]. To the best of our knowledge, no previous study has investigated the experiences of older patients above the age of 80 years of the quality of healthcare in hospital and the critical period after hospitalization. Thus, this study explores their experiences of the quality of the health services in hospital and the first 30 days at home after discharge. Our findings may enhance understanding of older patients’ experiences of adapting to daily life at home after discharge. By exploring the underlying mechanisms through the lens of healthcare quality and patient-centered care, we hope to provide useful information to reduce the gap between policy and clinical work in healthcare for older people.

## Methods

This study was part of a larger project addressing cross-sectoral care transitions for older patients from specialized hospital care to municipal health and care services, including the first 30 days after discharge [25]. The first period following hospital discharge is a vulnerable time for patients [24], and their journey between healthcare sectors may provide important information that can be used to improve the quality of transitional care.

### Design

Based on a qualitative design we used a phenomenological perspective to explore and describe older patients’ subjective experiences [26]. We sought to understand the complexity of the participants’ view of their situation, and our goal was to rely as much as possible on their perspectives.

### Setting and participants

In Norway, healthcare is mainly state funded and accessible to all inhabitants. Hospitals are primarily responsible for acute and specialized healthcare services, and the municipalities provide healthcare suitable for early identification and follow up of somatic and mental health [27]. Home care services in Norway are administered by the municipality for people who require healthcare services for long or short periods as a result of illness, impaired health, old age or other factors [28].

We recruited a purposive sample of participants [29] from an acute geriatric ward at a large hospital in Norway. Acute geriatric wards are defined as wards with an independent physical location and structure run by a specialized multidisciplinary team with direct responsibility for the care of older people with acute medical disorders [30]. Patients unable to give informed consent were not included in the study. The inclusion criteria were that patients had to be aged 80 years or above, living at home prior to hospitalization and were to be transferred to one of the following services: short-term stay at a nursing home (intermediate care) or own home with health services from the municipality. The health professionals at the hospital ward identified patients who fulfilled the inclusion criteria. Twenty-two patients agreed to participate and 18 were interviewed after discharge from hospital. The reasons for subject dropout included patients who were readmitted to hospital before the interview (*n* = 3) or changed their mind about participation (*n* = 1). To describe the functional and mental characteristics of the participants (see Table 1), we conducted the 30s Chair Stand Test (CST). The 30s CST is a functional evaluation clinical test that measures lower body strength, which is related to demanding activities in daily life [31, 32]. The test can predict health status, survival and hospital costs [33]. In addition, we performed the Mini Mental State Examination (MMSE) to assess cognitive function [34].

**Table 1:** Patient characteristics

No	Age	Gender	Hospital days	Intermed. care unit Y/N	Medical diagnosis	Medication at discharge	30 s sit-to-stand	MMSE^**1**^	Life situation	Next of kin	No. of inter-views
P1	95	F	8	Y	Pyelonephritis Sepsis Dehydration Kidney failure Hypertension Hypoalbuminemia Malnutrition	Esomeprazol Calcigran forte Lactulose Zopiclone Omega 3 Selo Zok Amlodipine Multivitamin Ciproxin	6	29	Alone	Daughter	2
P2	90	F	10	Y	Acute cystitis *E. coli* infection Orthostatic hypotension Hypertension Contusion lower back Anxiety Constipation	Albyl E Esomeprazol Lescol Levaxin	Not able to perform owing to physical condition	19	Alone	Daughter	2
P3	88	F	6	N	Pneumonia Aortic stenosis Hypertension Folic acid deficiency	Candesartan Albyl E, Simvastatin Taflotan eye drops Folic acid Apocillin	8	29	With spouse	Sister	2
P5	96	F	7	N	Heart failure Hypertension	Selo Zok Triatec Burinex Somac	Not able to perform owing to physical condition	25	Alone	Daughter	2
P6	95	F	8	N	Heart failure Cognitive failure Biological aortic valve Malnutrition	Burinex Renitec Spirix Metoprolol depot Calcigran forte Loratadine Prolia Imovane Paracetamol Triobet Warfarin	4	25	Alone	Grand daughter	2
P7	98	M	7	N	Acute respiratory tract infection Tumor bladder Hydronephrosis Acute renal failure Acidosis	Albyl E Burinex Cipralex Remeron Monoket Natron Multivitamins Omeprazole Imovane Enaton	1	27	Alone	Daughter	2
P8	96	F	5	N	Aortic stenosis Orthostatic hypotension Pulmonary hypertension	Azopt eyedrops, Selexid	7	28	Alone	Daughter	2
P9	97	F	7	Y	Orthostatic hypotension Concussion Rib fracture	Albyl E Zanidip Paracetamol Lactulose Folic acid Laxoberal Nobligan	Not able to perform owing to physical condition	24	Alone	Son	2
P10	87	F	7	N	Sepsis Kidney failure Acute tubulointerstitial nephritis	Albyl E Paracetamol Selo Zok Hiprex Amlodipine Levaxin Somac Ciproxin	One interview (test taken in second interview)	One interview (test taken in second interview)	Alone	Niece	1
P11	82	M	5	N	Nonspecific jaw disease Acute kidney failure Magnesium deficiency Anemia Ulcerative colitis	Bicalutamide Folic acid Nexium Prednisolone Ursofalk Imovane Magnesium	4	27	With spouse	Wife	2
P13	91	F	5	N	Chronic atrial fibrillation Stokes–Adams syndrome Vitamin D deficiency	Bisoprolol Digoxin Eliquis Montelukast Ventolin Atrovent Vitamin D Symbicort, Ventolin	One interview (test taken in second interview)	One interview (test taken in second interview)	Alone	Niece	1
P15	86	F	11	N	Acute tubulointerstitial nephritis Drop foot Kidney stones Degeneration of macula and posterior pole	Omeprazol Calcigran Taflotan Timosan Trusopt Hiprex Folic acid Albyl E Ciproxin	9	30	Alone	Daughter	2
P16	89	F	6	N	Syncope and collapse Acute cystitis Orthostatic hypotension	Vagifem Florinef Calcigran forte Plavix Lipitor Cortison Paracetamol	Not able to perform owing to physical condition	Not able to take test at the time of interview	Alone	Neighbor	2
P19	82	F	7	N	Gout Congestive heart failure Spinal stenosis Chronic atrial fibrillation	Burinex Xarelto Letrozole Levaxin Nexium Seretide Paralgin forte Paracetamol Movicol Colchicine DauAllopur	Not able to perform owing to physical condition	30	With spouse	Daughter	2
P20	86	M	6	N	Benign paroxysmal vertigo Unspecified thrombocytopenia	Losec Albyl E Triobe	10	30	Alone	Son	2
P21	100	F	6	N	Streptococcal sepsis Erysipelas Kidney failure	Adalat oros Ovesterin Bisoprolol Calcigran forte Albyl E Lamictal Ery-max	One interview (test taken in second interview)	One interview (test taken in second interview)	Alone	Son	1
P22	88	F	7	N	Sick sinus syndrome Syncope and collapse	Calcigran forte Albyl E Targinact Imovane Hylo gel Triobet Paracetamol	Last interview was by telephone (test taken in second interview)	Last interview was by telephone (test taken in second interview)	Alone	Support person	2
P23	96	F	15	Y	Pneumonia Unspecified pelvic and abdominal pain Obstipation Acute heart attack Atrial fibrillation and atrial flutter	Lercanidipine Albyl E Pursennid Pantoprazole Selo Zok Eliquis Prednisolone Bronkyl loss.	4	27	Alone	Grand daughter	2

Mini Mental State Examination (MMSE) to assess cognitive function 34. Tombaugh TN, McIntyre NJJJotAGS: **The mini-mental state examination: a comprehensive review**. 1992, **40**(9):922–935

Participants ranged in age from 82 to 100 years old, with a mean age of 92 years. The characteristics of the patients are shown in Table 1.

### Data collection

Data collection took place between September 2017 and March 2018. A semistructured interview guide with open-ended questions was developed. The questions were based on established standards for quality in healthcare [8] (see Table 2). To refine the questions, two pilot interviews were conducted. The interviews were conducted one-on-one 1–2 weeks after discharge at each patient’s current residence. To capture their recent experiences from the actual transition and to obtain information about their experiences from the whole 30-day period, a follow-up interview was conducted approximately 30 days after discharge. Three participants were only interviewed once owing to their health status at the time of the second interview. The interviews lasted from 30 min to 115 min, and were performed by the first author of this article, who is an experienced physical therapist with long experience working with elderly patients. The interviewer was not involved in the treatment of the patients in the study. All interviews were audiotaped and transcribed verbatim by a professional transcriber. Participant names and other personal identifiers were removed from the transcripts.

**Table 2:** Interview guide

1	Describe your situation right now.
2	Describe your experience of the hospital stay. a. Describe whether anyone at the hospital asked you what mattered most to you from your point of view. b. Describe your experience of the cooperation/coordination between the healthcare personnel at the hospital.
3	Describe the discharge process as you experienced it. a. Describe how/when you were informed about discharge. b. Describe the information you received about/during the discharge process. c. Describe whether any of the healthcare professionals asked you about your preferences regarding the discharge process. d. Describe whether your wishes regarding discharge were taken into account.
4	Describe your experience of the transfer between hospital and the healthcare services in the municipality.
5	Describe your experience of the cooperation/coordination between the personnel at the hospital and those in the healthcare services in municipality care.
6	Describe your experience of the information transfer between hospital and municipality.
7	Describe the time after discharge.
8	Describe your experience of the cooperation/coordination between the healthcare personnel in the municipality. a. Describe your experience of the cooperation between your GP and other healthcare personnel in the municipality.
9	Describe whether any of the healthcare professionals in the municipality asked you what mattered most to you from your point of view.
10	(If rehospitalization is a theme: Describe the rehospitalization process.)
11	Looking back, is there anything that should have been done differently regarding the healthcare you have received?

In qualitative studies, 15 ± 10 participants is considered to be a sufficient sample size to obtain information about the research phenomena with a manageable amount of data [35]. We aimed to recruit 20 patients or to continue recruitment until we reached data saturation [29].

### Data analysis

We analyzed the data using thematic analysis [36, 37], a method for identifying, analyzing and reporting patterns in qualitative data. Rather than starting with a theory, we inductively developed patterns of meaning through thematic analysis [38].

To ensure consistency of data analysis, we adopted the six-phase approach to thematic analysis described by Braun and Clarke [36]. This approach has been widely used and accepted as robust across a wide range of disciplines, including human health research [39]. To maximize trustworthiness and limit threats to validity, we employed the criterion for ‘trustworthiness’ outlined by Lincoln and Guba (1985) [40]. We satisfied the criterion of credibility through open-ended questioning, prolonged engagement with the data and by providing a detailed description of the methods. We fulfilled the criterion of transferability by presenting detailed and in-depth descriptive data and by quoting the participants. To satisfy the criterion of dependability, reiterative reading of the transcripts by two of the authors (I. L. and A. B.) was performed to transform the ideas generated into a set of codes to identify the interesting features of the data. These initial codes were then categorized into potential themes. The themes were discussed and reviewed by all authors to reflect on their relevance to the research questions [41]. All the authors who performed the analysis were educated in the health fields of physiotherapy (two), nursing (one) or nutrition (one), and have extensive research and/or clinical experience in the field of elder healthcare. The themes were then refined to ensure that each was meaningful and clear but distinct from other themes [42]. See Table 3 for an example of the coding strategy. In addition, we followed the consolidated criteria for reporting qualitative studies (COREQ) [43] (see the Supplementary File).

**Table 3:** Examples of coding strategy

Quotation	Initial code	Subtheme	Theme
*The hospital stay was lovely. I enjoyed it, I must say. I basically have nothing to put my finger on when it comes to nursing and care and such. I must say I was really happy with that stay, to come to a place where I could relax.* (P3)	The hospital stay was pleasant and a break from personal worries	The hospital as a place free from daily worries	Hospital stay and the person behind the diagnosis
*Then they took samples both here and there, and all kinds of examinations. And the next day it was the same lesson again with some blood tests.* (P6)	Many blood tests and different examinations	Biomedical approach	
*Yes, they told me in the morning because I hadn’t been told before. Therefore, I first thought that there would be no discharge that day either. But then they came and said, “You’re leaving now. You’ve got a room.” “Yes, well,” I just said then.* (P23)	I was not informed of discharge	Scant information	Poor communication and coordination
*I have no information about what is going on. No idea. I do not think there has been any cooperation between the hospital and the municipal health service. I do not think so.* (P7)	No cooperation experienced between the hospital and the municipality	Poor coordination	
*Right now, I am not very optimistic about my own situation, because I have been to the hospital and have the problems I have. I think that in time I must move to a retirement home. That’s probably what’s going to happen.* (P5)	I am pessimistic and insecure regarding my future	Insecurity	Life after discharge
*Now I have lost my courage. I’m too slow, I’m too tired, I have no strength for anything and then I get discouraged.* (P10)	I have no strength to handle everyday life and it makes me discouraged	Loss of ability to manage activities of daily living	
*My family is so helpful and I my neighbor is also very kind to me. She and her daughter buy groceries for me every Tuesday and that’s very helpful too.* (P21)	My family and my neighbor help me with everyday tasks	I could not manage without their help	Relationships with next of kin
*This is a little bit difficult for my kids to understand, you see, because they are used to me being able to manage by myself. They don’t quite understand this new situation. Because they haven’t seen me so helpless before, I think. They haven’t seen it. So now they see me as a more helpless person.* (P15)	I am helpless and need more help than before. It’s hard for my children	I am helpless	
*Yes, they* (the health personnel) *are busy. And that very day, I saw her running from one to the other. They must be tired. They must be.* (P20)	The health personnel are busy and do not have enough time	The health personnel have limited time to perform health services	Organizational and systemic determinants
*It’s not that bad now, I think. It’s been worse. I usually write down who’s coming, because I don’t always remember well. For example, Friday, October 6—Then one, two, three, four, five, six, seven, eight, nine different people came from the home service.* (P19)	Up to nine different people come each day	Different personnel came on each home visit	

### Ethical considerations

The study was preapproved and registered by the Norwegian Center for Research Data (No. 53110). All of the patients were deemed able to give informed consent by healthcare personnel in hospital. The first author approached the participants in hospital, and provided them with verbal and written information outlining the purpose of the study. We informed them that participation was voluntary and about their right to withdraw from the study at any stage. We assured them that this would not affect their current or future access to services. We obtained written and informed consent from all participants and guaranteed their confidentiality.

## Results

Our findings illustrate that the patients found their encounters with the health service to be complex and demanding. They reported that after discharge, they attempted to restore a sense of security and meaning to everyday life, balancing their own needs against external requirements, such as organizational and political demands. Below, we will present our findings in terms of five overarching and interrelated themes emerging from the interviews.

### Hospital stay and the person behind the diagnosis

The patients found the hospital to be a pleasant break where they felt safe and free from personal concerns. Most of the patients described the health personnel in the hospital as “*compassionate”* (P8), *“helpful”* (P22), *“respectful”* (P20) and *“nice”* (P5). Some of the patients had the opportunity to participate in and describe their subjective experiences of their illness: *“Yes, I was welcomed and listened to. I was a bit impressed by that”* (P8). Despite such experiences, most of the patients reported not feeling encouraged to be involved in collaborative decision-making, and not being asked about their preferences or needs. When questioned about whether the health personnel at the hospital had asked for their thoughts on their situation, what mattered most to them or what challenges they would meet at home, most answered along the lines of *“No, I don’t think so. I can’t remember them doing that”* (P21).

All of the patients reported detailed biomedical examinations: “*I’ve never been examined like that before in my whole life. It was so thorough, from head to toe. I thought it was fabulous. I have to say that I was speechless. I’ve never experienced such an examination before. They examined my lungs, heart, head and everything. And kidneys. It was a kidney specialist; he looked at the x-ray pictures, and there was another doctor for the lungs*” (P1). The medical examination made the patients feel safe and able to trust the expertise of the health personnel, even though little effort was invested in the process of getting to know the patient or searching for a solution to the health challenges that concerned them most: “*They didn’t talk to me about my shingles. They were not interested in that at all. They probably didn’t think it was the reason for my blood samples. They are more interested in tangible things. Things that they can investigate and take pictures of—things they can do something about*” (P15). Some of these examinations even resulted in new concerns for the patients: “*They were hung up on my kidney stones. I hope they don’t do anything about it, because they haven’t bothered me at all*” (P15).

### Poor communication and coordination

Although the participants were grateful for admission to hospital, most patients reported receiving scant information and too little time for preparation regarding the transition back home. As the following statements indicate, the departure came abruptly and unexpectedly to the patients: *“The days went so fast. Suddenly I had to go home. I just got the notice the same day”* (P20).

The patients’ experiences of communication and collaboration between the hospital and the healthcare services in the municipality regarding their return home varied. Most reported that the healthcare services in the municipality were informed and prepared for the patients’ transition, whereas others reported poor communication and failure to coordinate healthcare when they were transferred between levels of the healthcare system: *“No one prepared me for leaving the hospital and returning home. And back home—no plans for anything”* (P11).

Some patients in our study appealed for more information during their discharge process: “*So I thought maybe I should have some papers with me? I didn’t receive any information about anything. I found that strange too. I knew I was going home, but there was no information about any possible consequences of my disease, if there was something I should think about when I came back home, for instance, what to eat, and how to take medication and stuff. Nothing at all*” (P19).

Several of the patients experienced a lack of clear and comprehensible information. They described how written information contained terminology *“full of foreign words”* (P7), with a *“professional language* [that was] *not understandable”* (P7) to them. They also experienced how the written epicrisis created insecurity and new concerns: “*They gave me this huge journal, you know. It said something about me getting an ultrasound of the heart. Maybe that’s what I’m going to do in September? Maybe it’s heart failure? It’s hard to say. No one at the hospital said anything to me about heart failure. I don’t know if they examined my heart in the hospital. But maybe they did, because they took so many samples of everything, but I didn’t hear anything about heart failure. Because of what’s in written in the journal, I and my daughter have talked a little about the possibility that it is the heart. We talked about it, but there’s nothing we can do about it anyway”* (P5).

The patients in our study described being left in the dark with no opportunity to participate: *“They don’t ask me if it’s okay, they just tell me what to do. We’re not allowed to have an opinion”* (P2)*.* However, several of the patients accepted that the decision on further healthcare was taken by the healthcare personnel, because the patients felt that the health professionals know best: “*It’s okay that they decide. Because you are so tired. And, after all, it is the doctors’ decision”* (P23).

### Life after discharge

After discharge, the patients described a situation characterized by uncertainty and unpredictability. They describe life as a *“struggle”* (P3)*,* which they hope “*will be better”* (P3) in time. For some of the patients, a lack of information about unresolved health problems was the origin of the uncertainty.

This group of patients, with multiple chronic diseases and high degree of frailty, reported that their struggle was caused by loss of ability to manage the activities of daily life independently. They have neither “*the strength*” (P6) nor “*the energy*” (P20) to initiate daily activities. The patients described challenges related to house cleaning, grocery shopping and social participation. They narrated how they struggled to participate in meaningful activities, primarily owing to their physical condition: *“Previously I had guests all the time, but I don’t have the same capacity anymore. I have realized that. I just hope I can get a little bit better. I can’t do things the way I did before. It is no use. It has gone steeply downwards with my social life”* (P15). A more isolated social life led to loneliness, depression and a feeling of despair and nothing to live for: *“I have missed out on so many beautiful days this summer. I am so tired of this life. In two years, I will be one hundred. I certainly don’t want to get that old. Especially when I have become as disabled as I am now”* (P7).

Most of the patients strove to maintain their independence and master everyday tasks. They had to rely on help from relatives and healthcare professionals to manage life at home. They described the situation using words such as *“helplessness”* (P13)*, “desperation”* (P13)*, “concern”* (P6) and *“fear*” (P7). Because of the challenges they encountered at home, and the insecurity and uncertainty that characterizes their situation, the thought of *“giving up”* (P19) had crossed their minds.

### Relationships with next of kin

An informal caregiver, ranging from a spouse to an adult child/grandchild, niece, friend or neighbor, usually assisted the patients with needs beyond those that the health services could support. The patients describe the help as a necessary resource to cover practical needs. Almost all of the participants in this study received regular help with grocery shopping from family or neighbors. Laundry, house cleaning, transportation and paying bills are other examples of needs covered by the participant’s next of kin. The patients expressed the view that they would not be able to manage everyday life without help from their caregivers. Quotes such as *“My son organizes everything for me”* (P9) and *“I don’t understand how I could manage without their help”* (P23) illustrate how dependent they are on this help*.* They also describe how relatives act as a safety net in the time after hospitalization, and the patients trust that their next of kin will take appropriate action if they become sicker or if the home situation becomes *“unsafe”* (P20).

The patients emphasize positive and strong relations with their next of kin as an important factor in managing life after discharge. However, they worried that their illness would put a strain on these relationships. The patients were aware that informal caregivers had *“limited time owing to other commitments”* (P10)*.* Several of the patients reported feeling like a burden on their closest relatives in the period after hospitalization: *“I’m just a burden to everyone around me. Everyone has to take care of me. It doesn’t feel good. I’ve always been able to manage by myself”* (P13). This concern resulted in feelings of stress, anxiety and guilt.

### Organizational and systemic determinants

Many of the patients reported that the workload and time pressure on health personnel affected the healthcare they received. The patients blamed the system rather than the staff, and they accepted that health professionals performed their clinical work while balancing patient needs, available resources and regulatory constraints. The participants found that the healthcare personnel in the hospital and in the municipality had limitations: *“They are probably doing their best. But they’re always in a hurry. It is quite clear. They are so frantic, rushing from one person to another. So they take advantage of the staff”* (P2).

Four of the 18 patients were transferred to intermediate care units before returning to their own homes. These patients found the healthcare staff in intermediate care to be stressed, distant or in a hurry, with “*little time to speak with me”* (P1). They also reported that the professionals in intermediate care wards had little ability to offer individualized care to patients, owing to limited resources*: “The days get long in here. The nights as well. I wake up very early, usually at half past five or six. But I have to stay in my bed until 8.30 or 9 am. They don’t have time before that. They don’t start work so early, either. So I rarely get out of bed before nine. They are very busy”* (P2).

Most of the patients receiving healthcare services from the municipality reported short visits. These allowed little time for conversation or discussions of matters of concern to the patients. The patients also found that different personnel came on each visit, and several reported extensive use of temporary workers: *“There are different people (from the homecare service) each time. It’s never the same person”* (P5).

Several of the patients commented that their general practitioner was very busy, with too little time for them. They described visits with a biomedical focus, where the patient’s own thoughts about his/her illness were not a theme; nor was how the disease had affected the patient’s daily life. The following quotes illustrate patients’ experiences of meetings with their general practitioner.

*I noticed (that the GP was in a hurry), because she started talking about other things, getting up and moving towards the door, because she was 1 hour late. I was about to ask about other things, but then I didn’t do that.* (P6).

*The GPs have a lot to do, are in a hurry. They don’t have time for the patient. They have to write things and look at the screen with no attention given to the patient. But that’s how it is. They probably have around 15 min per patient.* (P15).

## Discussion

The results of this study indicate that elderly people receiving healthcare in hospitals and in the municipality face several challenges regarding the quality of services. The elderly patients reported few invitations to participate in decisions regarding their own care, to share their concerns or to tell staff members what was important to them. They reported insecurity and distress owing to a lack of information and coordination between the levels of the healthcare system. They described limitations in their capacity that made daily tasks challenging and prevented them from participating socially. The caregivers helped patients with practical needs when the health service was unable to assist owing to organizational and systemic determinants. They received the needed help with gratitude, but also described a loss of autonomy, helplessness and a fear of being a burden to everyone. The patients experienced healthcare services that focused on their diagnosis rather than listening to their concerns or addressing their need for practical assistance and social participation.

The discussion below is organized according to the main factors in the WHO’s strategy for quality improvement. The WHO describes quality healthcare as services that are effective, efficient, integrated, patient centered, equitable and safe [8].

For effective care, the WHO states that the service should provide evidence-based care to those who need it [8]. The elderly patients in our study experienced a health service that seemed to be based on measurable values. The thorough biomedical examination and the focus on revealing a diagnosis and defects in the elderly patients may be an expression of a desire to ensure that the services are consistent with knowledge about and evidence for achieving the best possible outcomes for the patients [3]. The biomedical model of the past century has been valuable for some aspects of medicine, and is a necessary but not sufficient component in the proper care of patients [44]. Modern medicine has successfully applied biomedical science through a model that defines disease as the disruption or deviation of biological variables. This focus on deviations from biological norms can lead to the neglect of patients’ needs, and the medical examination is seen as an intrinsic good in itself rather than as a means to help individuals achieve goals that are important to them [44]. This is in line with the patients’ experiences; few reported being asked about their goals or what mattered to them. Failure to honor the patients’ goals can result in the overuse of disease-focused treatments at the end of life [44]. The patients in our study reported that detailed examinations resulted in new diagnoses, which led to further medical investigations. On the other hand, the thorough medical examination made the patients feel safe and made them trust the competence of health personnel. Delivering safe services is one of the main components of quality healthcare [8].

In addition to the health services being effective and safe, according to the WHO, they must also be patient centered [8]. Patient centeredness represents a cultural shift in healthcare [45] from the biomedical model, in which the patient is a passive target of the healthcare system, to a model where the patient is an active partner in his/her care and decision-making [45, 46]. As indicated above, the patients interviewed did not experience this shift. Instead, they reported that the services were concerned about examining them, diagnosing them and treating their diseases. Few patients in this study were included in clinical decisions regarding their own health. Some of our participants even reported that they were not asked or allowed to have an opinion about their own healthcare. Despite decades of attention to principles of activating, empowering and engaging the patients in their own healthcare [9, 47–49] as part of securing patient centeredness as a cornerstone of healthcare service quality [48, 50], the service did not appear to be responsive to older patients’ preferences or needs.

Patient centeredness and patient-centered care often assumes that all patients have the competence and ability to participate in important decisions about their own care [51]. The frameworks for participation are often characterized by efficiency requirements, so patient groups with reduced physical and cognitive abilities may have limited opportunities to participate [52, 53]. Our patients reported reduced capacity with little capability to engage in daily tasks and decisions concerning their own health. The decision-making capacity of older patients may be limited owing to cognitive decline, emotional distress, multiple chronic conditions or a combination of these [54]. Shared decision-making with older patients who are frail requires a continuous counselling dialogue [55] adapted to each patient’s capacity. Equitable care for this patient group means that elderly people have the same rights to healthcare as everyone else [8], but it also means that healthcare authorities and personnel have an obligation to make particular efforts to secure healthcare attuned to older patients’ needs and preferences [3]. Our results confirm the findings of other studies that older adults with multiple chronic conditions receive care that is fragmented and seldom focused on what matters most to the patient [56].

Current policies in the Western world emphasize that good quality in healthcare is related to older people living in their own homes. It is also documented that most older people prefer to age in their own home [57, 58]. Consistent with this, the overall aim of home care services is to assist older people to live independently in their homes and maintain quality of life for as long as possible [59]. However, our findings indicate that the elderly patients experienced significant problems in maintaining quality of life and independence after discharge. Multimorbid or frail patients may require services that are not frequently considered part of the clinical routine (e.g., support at home for daily activities and transportation) but are crucial for the success of clinical interventions for this patient group [60]. According to the patients, the gap between their need for assistance to cope with everyday life after discharge and the help provided by the services in the municipality had to be filled by family and relatives. This finding agrees with previous research, which highlights that to rationalize limited resources, relatives are important but often invisible contributors to elderly patient care [61, 62]. In line with previous research, the patients in this study also relied on family and informal caregivers for their social needs [63, 64]. Being dependent on help from caregivers and friends was a concern for the patients. They reported that dependence led to concerns about being a burden on their loved ones. Other studies showed similar results, describing older patients worrying about burdening their families because of practical matters such as time and resources [65]. For our participants, being a burden to others was associated with stress and anxiety. Some studies showed that for patients, the sense of being a burden is related to depression, distress and reduction in life quality [66, 67]. By not receiving equitable care adapted to their health conditions and their needs, the patients in this study were left in limbo, struggling to find a balance in their everyday life.

Providing efficient services means that the resources are allocated and used in the best possible manner to achieve desired outcomes [8]. The patients encountered healthcare personnel with limited time. Most of the patients receiving healthcare services from the municipality reported short visits with little time for conversation or discussions of what mattered to the patient. The patients blamed the system rather than the staff, and they accepted that health professionals in their clinical work balanced the patient’s needs against available resources and regulatory constraints. Health personnel are often constrained by the goals, requirements and limitations imposed by organizational and systemic determinants, and they must find ways to resolve the possible incompatibility between patient-centered goals and organizational goals [68, 69]. Thus, the healthcare quality experienced by older patients is limited by organizational constraints and the dilemmas that health professionals must address in their work. As people with multimorbidity face multiple health and/or social problems, it is especially important to offer a patient-centered integrated care approach that is tailored to the individual and his/her environment. The situation of people with multimorbidity may change over time, so flexibility is important—flexible care can be continuously updated to match a person’s needs [70]. Based on the patients’ experiences, it appears that healthcare services, owing to organizational determinants, fail to deliver flexible patient-centered healthcare attuned to issues of significance to elderly patients. Research has shown that individualized patient-centered interactions promote adherence to treatment and lead to improved health outcomes [71]. By receiving personalized services, the patients may increase their capacity and live independent lives in own homes at reduced cost of help and care.

Quality of healthcare means providing care that is integrated and coordinated across levels [8]. For this group of patients, quality of healthcare services depends greatly on good planning to provide a safe journey through the healthcare system and reduce readmissions to hospitals [70, 72]. This process includes effective collaboration and communication between patients, their carers/next of kin and health professionals when moving across care settings [17, 73]. As mentioned above, elderly patients are being discharged from hospital “quicker and sicker” than before [5, 6, 74], leaving the healthcare provider with limited time to coordinate services across settings [7]. Poor communication and suboptimal care coordination [72] across healthcare providers and settings produce more frequent hospital readmissions and poor quality outcomes [75]. Despite focus in recent years on improving the coordination of care across care settings, our findings show that communication across care settings still needs improvement. Lack of integration between the levels in the healthcare system left the patients feeling unsafe and insecure and caused them concern about their future health.

### Strengths and limitations

As in other qualitative research, the goal of this study was to enhance our understanding of the phenomenon being studied [26]. We aimed to give a voice to older patients about their experiences of the quality of healthcare services. However, the study is limited by the specific demographics of the small sample size and the geographical location. Even though the findings in this study cannot be generalized, the results may be transferred to similar situations or people [26].

The authors of this study have backgrounds in nutrition, nursing and physiotherapy, in addition to clinical practice, leadership, quality improvement and professional development in the health services. Throughout the study, we were conscious that our previous understanding and backgrounds would influence the research process [29]. We strove to interpret the data openly and to provide a transparent description of the path from the data to the results. The involvement of multiple researchers from different backgrounds may strengthen the design of a study, as they can supplement and contest each other’s statements [76].

The patients’ vulnerable situations and poor health may have influenced the quality of the interviews. The interviewer did her best to put the participants at ease and to listen empathetically and carefully, and several patients expressed their appreciation of the opportunity to tell their story. By conducting a follow-up interview, we were able to grasp the situations and experiences throughout the 30-day period. This may be a strength of the study.

## Conclusion

To deliver healthcare quality, all of WHO’s six dimensions (effectiveness, efficiency, integration, patient centeredness, equitableness, safety) must be present. The elderly patients’ stories shows us that these dimensions are not always present. Instead of focusing on the special needs of elderly patients with multimorbidity, the healthcare services were attuned to measurable values and biomedical inquiries. Few possibilities for participation, scant information and suboptimal care coordination left the patients with a feeling of being in limbo, where they struggled to find balance in their everyday life.

This study in line with other research, shows that further work must be done to ensure coverage of integrated services without a financial burden, centered on the needs and rights of older people [54]. Healthcare quality for elderly people demands a healthcare system organized in ways that ensure care and support consistent with their basic rights, fundamental freedoms and human dignity.

### Relevance to clinical practice

Elderly people have a right to make choices and take control over a range of issues including their own healthcare. The possibilities for choice and control are shaped by many factors, including the intrinsic capacity of the older people, the environments they inhabit, the personal and financial resources they can draw on, and the opportunities available to them. Together these determine the autonomy of older people, which has been shown to have a powerful influence on their dignity, integrity, freedom and independence, and has been repeatedly identified as a core component of their general well-being.

To deliver effective, efficient and integrated healthcare to older patients, the service needs to be perceived as patient centered, equitable and safe by the patients themselves.

To achieve this, the healthcare services must:

- shift their focus from disease to intrinsic capacity
- be organized in ways that secure person-centered and integrated care for older people
- ensure there are highly qualified personnel with expertise in the needs and challenges that older patients with multimorbidity face in healthcare and in daily life.

## Supplementary information

**Additional file 1:** COREQ checklist.

## Data Availability

The datasets generated and/or analysed during the current study are not publicly available due to privacy concerns but are available from the corresponding author on reasonable request.
